# Biofilm Formation by *Pseudomonas aeruginosa* in a Novel Septic Arthritis Model

**DOI:** 10.3389/fcimb.2021.724113

**Published:** 2021-09-21

**Authors:** Dingbin Li, Li Zhang, Jinhua Liang, Wusheng Deng, Qingjun Wei, Ke Wang

**Affiliations:** ^1^Department of Orthopedic Trauma and Hand Surgery, The First Affiliated Hospital of Guangxi Medical University, Nanning, China; ^2^Department of Pulmonary and Critical Care Medicine, The First Affiliated Hospital of Guangxi Medical University, Nanning, China

**Keywords:** septic arthritis, *Pseudomonas aeruginosa*, biofilm formation, cyclic di-GMP, animal model

## Abstract

**Background:**

Bacterial biofilms generally contribute to chronic infections and complicate effective treatment outcomes. To date, there have been no reports describing biofilm formation in animal models of septic arthritis caused by *Pseudomonas aeruginosa* (*P. aeruginosa*). *P. aeruginosa* is an opportunistic pathogenic bacterium which can lead to septic arthritis. The purpose of this study was to establish a rabbit model of septic arthritis caused by *P. aeruginosa* to determine whether it leads to biofilm formation in the knee joint cavity. In addition, we explored the role of cyclic di-GMP (c-di-GMP) concentrations in biofilm formation in rabbit models.

**Methods:**

Twenty rabbits were randomly assigned to five groups: PAO1 (n = 4), PAO1*ΔwspF* (n = 4), PAO1*/p_lac_-yhjH* (n = 4) infection group, Luria–Bertani (LB) broth (n = 4), and magnesium tetrasilicate (talc) (n = 4) control groups. Inoculation in the rabbit knee of *P. aeruginosa* or with the same volume of sterile LB or talc in suspension (control group) was used to induce septic arthritis in the animal model. In the infection groups, septic arthritis was caused by PAO1, PAO1*ΔwspF*, and PAO1*/p_lac_-yhjH* strains, respectively. Rabbits were euthanized after 7 days, and pathological examination of synovial membrane was performed. The biofilms on the surface of the synovial membrane were observed by scanning electron microscopy, while the biofilms’ fiber deposition was discriminated using peptide nucleic acid-fluorescence *in situ* hybridization (PNA-FISH).

**Results:**

A rabbit model for knee septic arthritis induced by *P. aeruginosa* was successfully established. Scanning electron microscopy revealed that PAO1 strains were surrounded in a self-produced extracellular matrix on the surface of synovial membrane and showed biofilm structures. The biofilms in the fibrous deposition were also observed by PNA-FISH. The PNA-FISH assay revealed that the red fluorescence size in the PAO1*ΔwspF* group was greater than in PAO1 and PAO1*/p_lac_-yhjH* groups.

**Conclusions:**

This is the first study to provide evidence that *P. aeruginosa* forms biofilms in a rabbit model for septic knee arthritis. The rabbit model can be used to investigate new approaches to treatment of biofilms in septic arthritis. Furthermore, c-di-GMP is a key signaling molecule which impacts on biofilm formation in rabbit models of knee septic arthritis.

## Introduction

Septic arthritis is an invasive disease that can lead to wide articular cartilage and bone defects and irreversible impairment of joint function (Chan et al., 2020; Margaryan et al., 2020). The incidence of septic arthritis among the general population is about 4 to 12 cases per 100,000. Furthermore, there is a high (10%–15%) fatality rate among septic arthritis patients (Kennedy et al., 2015; Abram et al., 2020). Septic arthritis also presents subjective adverse results in 20%–30% of patients due to its potential life-threatening symptoms, and the commonest joint infected by septic arthritis is the knee (Abram et al., 2020). The poor response to treatment is associated with the lack of information regarding disease pathogenesis.

The most common pathogen of septic arthritis is *Staphylococcus aureus* (Baranwal et al., 2017). However, *Pseudomonas aeruginosa (P. aeruginosa)*, a Gram-negative bacterium, comprises a large portion of the pathogens causative of septic arthritis (Shirtliff and Mader, 2002). *P. aeruginosa* strains are ubiquitous yet are most well-known for their effects in chronic infections, and they grow as biofilms, which results in high rates of antibiotic resistance, resulting in morbidity and mortality (Botelho et al., 2019; Pang et al., 2019). Biofilms are bacterial communities that adhere to biological or abiotic surfaces and are encased in a self-produced highly hydrated matrix (Cendra and Torrents, 2021). Some studies have reported that most microorganisms are more likely to live in biofilms rather than in planktonic cultures (Costerton et al., 1978; Donlan and Costerton, 2002). These sturdy communities result in a wide variety of adverse effects in many aspects of our daily life, yet it is a significant challenge to eradicate them because biofilms remain incredibly resistant to conventional antimicrobial agents (Blackman et al., 2021; Cendra and Torrents, 2021). Biofilm development on living surfaces has been reported in keratitis (Saraswathi and Beuerman, 2015), empyema (Zhang et al., 2020), tonsillitis (Kania et al., 2007), and otitis media (Hall-Stoodley et al., 2006). Although a case report has recently found that infections by *P. aeruginosa* in the wrist joint were related to biofilm formation (Thibeault et al., 2020), there has been no evidence to date that septic arthritis forms biofilms, which may explain the resistance to therapy. We speculate that *P. aeruginosa* may form biofilms in the joint cavity of septic knee arthritis. Biofilm therapy is influenced by many factors. Constructing a septic arthritis model of biofilms may contribute to identifying mechanisms involved in biofilm formation and antibiotic efficacy to achieve good treatment results.

Cyclic di-GMP (c-di-GMP) is an important second messenger of intracellular signal transduction in many bacteria controlling motility, biofilm formation, and virulence (Jenal et al., 2017; Valentini and Filloux, 2019). Usually, high intracellular c-di-GMP content enhances biofilm formation, while low c-di-GMP content leads to biofilm dispersal (Ha and O'Toole, 2015; Jenal et al., 2017). In *P. aeruginosa*, intracellular c-di-GMP has the key function of modulating biofilm formation and antibiotic efflux pump expression (Wang et al., 2018; Cendra and Torrents, 2021). Despite numerous studies investigating c-di-GMP signaling since the 1980s (Ross et al., 1987), the identification of the triggers stimulating diguanylate cyclases and c-di-GMP-specific phosphodiesterases remains unclear, and most signaling domains expressed by these activator proteins have no known function (Zarrella and Bai, 2020). Therefore, constructing an *in vivo* model of biofilms is important for investigating the mechanism of biofilm formation.

Previous studies have not described biofilm formation in animal models of septic knee arthritis. The purpose of this study is to design and construct a *P. aeruginosa* biofilm model of septic knee arthritis in a rabbit model, to observe the morphology and complex spatial structure of biofilms using electron microscopy and PNA-FISH. In addition, we explored the effects of intracellular c-di-GMP levels on biofilm formation in the rabbit model. The construction of a *P. aeruginosa* biofilm model is of great significance for the in-depth study of mechanisms involved in *P. aeruginosa* biofilm formation.

## Materials and Methods

### Animals

Twenty clean-grade 3- to 4-month-old healthy New Zealand white rabbits, weighing 2–3 kg, of both sexes provided by the Experimental Animal Center of Guangxi Medical University were used in this study. We obtained animals 1 week before the interventions and allowed them to adapt to the housing conditions. Rabbits were maintained in separate cages. Rabbits were fed with water and 80–100 g of antibiotic-free rabbit food at will. Feeding conditions included room temperature 18°C–25°C, air circulation, and relative humidity 40%–70%. Twenty rabbits were assigned to five groups according to a random number table: three experimental groups (PAO1, PAO1*ΔwspF*, PAO1*/p_lac_-yhjH* infection group) and two control groups (Luria–Bertani [LB] broth, magnesium tetrasilicate (talc) group); each group comprised four rabbits. All animal experiments were reviewed and approved by the Medical Ethics Committee of the First Affiliated Hospital of Guangxi Medical University (no. 202007001).

### Preparation of Bacterial Inoculum

*P. aeruginosa* strains: a PAO1 wild-type strain, a *ΔwspF* mutant characterized by high intracellular c-di-GMP levels, and a PAO1*/p_lac_-yhjH* mutant characterized by low intracellular c-di-GMP levels were provided by Singapore Centre on Environmental Life Sciences Engineering, Nanyang Technological University, Singapore (Chua et al., 2013; Chua et al., 2015). The strains were preserved in LB (LB, Guangdong Huankai Microbial Technology Co., Ltd., Guangzhou, China) broth containing 25% glycerol at −80°C. LB with 60 μg/ml tetracycline was used to maintain the plasmid of the PAO1*/p_lac_-yhjH* strain. We used a sterile inoculation loop to select an appropriate amount of frozen-preserved strain, which was inoculated onto the LB agar plates (LB Agar, Beijing Land Bridge Technology Co., Ltd., Guangzhou, China) and placed in a 37°C incubator (HPX-400, Shanghai Yuejin Medical Equipment Co., Ltd., Shanghai, China) for 24 h, and subsequently transplanted into LB liquid medium and incubated on a rotary shaker (THZ-82, Changzhou Zhibo Instrument Manufacturing Co., Ltd., Changzhou, China) for 18 h at 37°C, at 220 rpm. The bacterial solutions were centrifuged (3,000 rpm for 15 min) (Z 366, HERMLE Labortechnik GmbH Co., Ltd., Germany). Any residual media components were removed, and strains were thoroughly washed with phosphate buffer solution (PBS) three times. *P. aeruginosa* strains were suspended in LB, and the final concentration of strains was measured with a spectrophotometer (UV-Visible Spectrophotometer T6, Beijing Purse General Instrument Co., Ltd., Beijing, China). We defined the optical density (OD) value of 0.1 as 2 ml LB with 10^8^ colony-forming units (CFU)/ml, and diluted aliquots to 10^6^ CFU/ml with LB (Zhang et al., 2020).

### Measurement of c-di-GMP Content and Comparison of Biofilm Formation *In Vitro*

*P. aeruginosa* strains—PAO1/p_cdrA_ *-gfp* containing the p_cdrA_ *-gfp* vector, PAO1*ΔwspF*/p_cdrA_ *-gfp* containing the p_cdrA_ *-gfp* vector characterized by high intracellular c-di-GMP levels, and PAO1*/p_lac_-yhjH*/p_cdrA_ *-gfp* containing the p_cdrA_ *-gfp* vector characterized by low intracellular c-di-GMP levels—were provided by the Singapore Centre on Environmental Life Sciences Engineering, Nanyang Technological University, Singapore (Chua et al., 2013; Chua et al., 2015). For plasmid maintenance of PAO1/p_cdrA_ *-gfp* and PAO1*ΔwspF*/p_cdrA_ *-gfp*, the medium was supplemented with 50 µg of carbenicillin/mL. For plasmid maintenance of PAO1*/p_lac_-yhjH*/p_cdrA_ *-gfp*, the medium was supplemented with 50 µg of carbenicillin/mL and 10 µg of tetracycline/mL. According to the method described above, 2 ml of bacterial suspension with OD value of 0.1 was obtained, and then 200 μl was transferred to each well of a 96-well plate. Six wells were repeated for each group. After incubation in a biochemical incubator at 37°C for 24 h, the optical densities of 600 nm (OD600) and green fluorescent protein (GFP) fluorescence (in relative fluorescence units) (emission wavelength 535 nm; excitation wavelength 485 nm) were recorded for each well of a 96-well microplate. The relative fluorescence intensity (RFI) was calculated by dividing the GFP value by the OD600 value (Chua et al., 2013). The experiment was repeated three times.

To measure the expression of p_cdrA_ *-gfp* in biofilms, the *P. aeruginosa* pao1/p_cdrA_ *-gfp* strain was cultured in a 50-ml tube containing 15 ml LB medium (the bacterial solution concentration was 10^8^ CFU/ml with LB) according to a literature (Chua et al., 2013). A sterile glass coverslip (24 × 60 mm) was inserted into each 50-ml tube to support biofilm growth. After incubation in a biochemical incubator at 37°C overnight, the biofilms formed on the slides were washed twice with 1 ml of 0.9% NaCl and imaged with a fluorescence microscope (EVOS FL Auto 2, Invitrogen by Thermo Fisher Scientific, USA). The experiment was repeated three times.

*Biofilm formation test*: The biofilms were quantified as described in reference (Heinonen et al., 2021), and some modifications were made. In short, 200 μl of bacterial suspension with OD value of 0.1 was transferred to the well of a 96-well plate. After incubation in a biochemical incubator at 37°C for 24 h, biofilms were washed with PBS for three times and stained with 0.1% crystal violet (100 ml, Beijing Solarbio Science & Technology Co., Ltd., Beijing, China) at room temperature for 15 min. After washing the wells under mild water flow and careful drying, stained biofilms were dissolved in 33% acetic acid, and the absorbance was measured at 595 nm using a spectrophotometer (3020, Thermo Fisher Scientific Oy, Finland). The experiment was repeated three times.

### *P. aeruginosa* Septic Knee Arthritis Induction

Rabbits were placed in a rabbit surgery box and anesthetized by intravenous injection of 10% chloral hydrate (Chengdu Kelong Chemical Co., Ltd., Chengdu, China) (dissolved in water at a dose of 5 g/50 ml) at the ear-edge vein in doses of 200 mg/kg. Rabbits were then moved to an operating table and placed in supine position to fully expose the surgical site in front of the right knee joint to perform routine preoperative preparation (skin preparation, disinfection, and laying of aseptic towels). After palpating the lateral edge of the patella, the proximal edge of the tibia, and the distal edge of the lateral femoral condyle, we used a 5-ml syringe to inject 2 ml of the abovementioned bacterial solution into the right knee joint cavity of each rabbit through the patellar tendon and injected 2 ml of LB or talc suspension (0.25 g talc in 2 ml physiological saline solution) for the two control groups. After regaining consciousness, rabbits were housed routinely for 7 days, and surviving rabbits were taken for inspection of various indicators. All experiments were repeated three times.

### Euthanization and Tissue Specimen Extraction

Based on a point system, clinical assessment was performed on day 7 post-inoculation; clinical grades were used to rate arthritis erythema and swelling on a scale of zero to three (Puliti et al., 2002). Results of the arthritis index were expressed as mean ± standard deviation (SD). On day 8, all rabbits were euthanized by a lethal dose of chloral hydrate. Preoperative preparation was the same as mentioned above. A small median linear skin incision was made in front of the knee of 6 cm in length. The morphology of the gross anatomy of the knee cavity was observed and photographed after removing the skin, subcutaneous tissue, superficial fascia, distal end of the patellar ligament, and proximal end point of anterior joint capsule: We collected all the fibrinous deposition of purulent exudate in the knee joint cavity, weighed them, put them in a 10-ml sterile centrifuge tube containing 10 ml PBS, which were then placed in an ice box, and centrifuged at 3,000 rpm for 15 min on returning to the laboratory. The sediment samples were preserved in a refrigerator (LSC-368C, Zhejiang Star Appliance Co., Ltd., Zhejiang, China) at 4°C until analysis. The synovial tissue of anterior joint capsule was excised, divided into two pieces, and stored in a specimen bottle filled with 10% formaldehyde solution or 2.5% glutaraldehyde solution for scanning electron microscopy (SEM), respectively. The synovial fluid was inoculated on the LB agar plate using a sterile inoculation loop and incubated at 37°C for 24 h.

### Tissue Colony Count

The fibrinous deposition was collected from each sample; 0.15 g was weighed and placed into a 2-ml sterile centrifuge tube. A 1-ml volume of sterile PBS solution was added to the centrifuge tube. After shaking gently, the wash ing solution was removed and 500 µl PBS solution was added. A homogenizer (sample freezing grinding instrument, Guangzhou Luca Sequencing Instrument Co., Ltd., Guangzhou, China) was used to homogenize tissues for 1 min (homogenizing speed and time: 70 Hz/s). The fibrinous deposition colonies of each sample were counted with a 10-times dilution method. Combined with the dilution multiple of the sample and the weight of each sample, the bacterial load of the fibrinous deposition in each sample was calculated and recorded as 1 g (CFU/mL).

### Histopathological Examination

The specimens were fixed in 10% neutral paraformaldehyde for 24 h and decalcified by 5% dilute hydrochloric acid for 12 h, then embedded in wax blocks and cut into 4-μm-thick sections for routine hematoxylin and eosin (H&E) staining; we examined each H&E-stained slide for histopathological changes and measured the thickness of the synovial membrane using an optical microscope, as previously described (Mohammad et al., 2019; Zhang et al., 2020). Briefly, we made a central section of the sagittal plane of the patella and photographed the anterior synovium of the knee for ×20 imaging with a microscope (Eclipse Ci-L, Nikon, Japan). After imaging, we took the micron as the standard unit and measured the thickness of the synovial hyperplasia at five places along each slice using analysis software (Image Pro Plus 6.0, Media Cybernetics, USA) and took the thickest value. A total of 20 samples were used for analysis.

### Scanning Electron Microscopy

The specimens for SEM were fixed with 2.5% glutaraldehyde solution at 4°C for 2 h, washed with PBS solution three times (1 min each time), immersed in 1% osmic acid solution at 4°C for 2 h, and dehydrated serially in 50%, 70%, and 95% absolute ethanol solutions for 10 min each. Risoamyl acetate replaced ethanol for dehydration steps for 20 min at 4°C. Finally, the sample tissues were dried in a vacuum, sprayed with an IB3 (IB5) ion-sputtering instrument, and observed through SEM.

### Peptide Nucleic Acid Fluorescence *In Situ* Hybridization (PNA-FISH)

In order to further observe and quantify the biofilm, we used the PNA-FISH method with a PNA-FISH kit (AdvanDx, MA, USA) (Xu et al., 2019). PNA-FISH was performed as described previously (Zhang et al., 2020; Castro et al., 2021): (1) dewaxing: the baked glass slides were soaked in xylene, twice, 5 min each time; (2) the slides were soaked in 99.9% ethanol, twice, 3 min each time, and then soaked in 96% ethanol, twice, 3 min each time; (3) the slides were soaked in pure water, three times, 3 min each time, and then dried at room temperature; (4) Texas Red-labeled *P. aeruginosa*-specific PNA probes were prepared: diluted Texas Red-labeled *P. aeruginosa*-specific PNA probes were prepared in the dark, centrifuged briefly, and suspended in 34 µl of RNase-free water, and serially diluted 10-fold serially to a 100-fold dilution followed by a 3-fold dilution, to achieve the working concentration required by the experiment of 330 nM; (5) a 10-µl drop of the diluted probe was added to the specimen, which was then covered by a cover glass, and any bubbles under the cover glass were expelled using a rubber and sealed; (6) the slides were placed in a dark humidity box in a 55°C incubator and hybridized for 90 min; (7) the rubber glue on the glass slide was removed together with the cover glass, and the slide was immersed in a preheated 2× SSC washing solution at 55°C for 30 min; (8) the slide was dried at room temperature, and a 10-µl drop of DAPI anti-fade counterstaining agent was added to the target area of the slide, and bubbles were expelled and stored in a dark place for 15 min, to be observed. Finally, a fluorescence microscope was used to measure the degree of the red fluorescence. After imaging, we used analysis software (Image Pro Plus 6.0, Media Cybernetics, USA) to measure the red fluorescence area in each slice. Each rabbit repeated the slice three times and selected the value of the maximum red area. Finally, a total of 20 samples were used for analysis.

### Statistics

Data was analyzed by SPSS v.25 software (IBM Corp., Armonk, NY, USA) using descriptive (frequency, mean, and standard deviation) statistics. For the comparison of multiple groups, a one-way analysis of variance (ANOVA) was utilized, and pairwise comparisons were conducted, and *p*-values of <0.05 were considered statistically significant.

## Results

To determine whether *P. aeruginosa* could induce biofilm formation in a rabbit model for septic knee arthritis, rabbits were inoculated with 10^6^ CFU of *P. aeruginosa* (2 ml) in the knee joint through intra-articular injection and were followed for up to 7 days.

### Macroscopic Appearance

**Figure 1:**
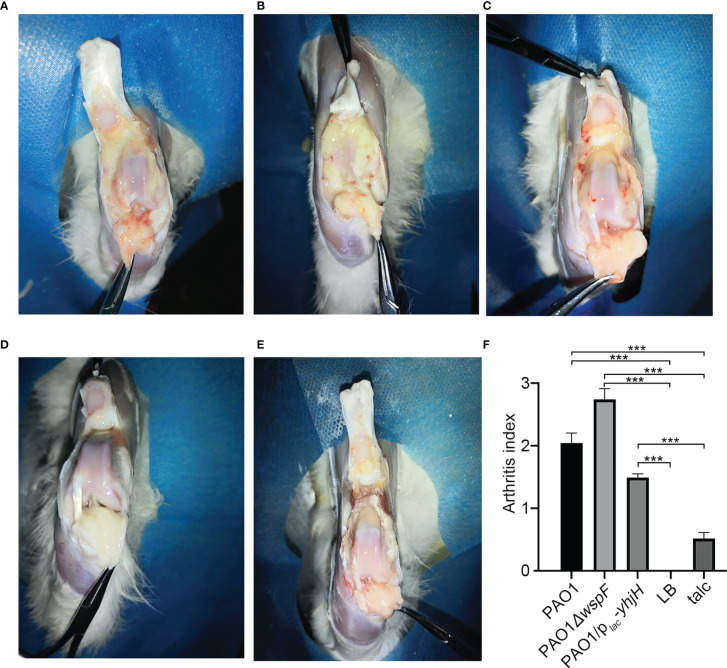
The gross pathology specimens of rabbit joint cavities 7 days after inoculation. **(A)** PAO1 group. **(B)** PAO1*ΔwspF* group. **(C)** PAO1/p*_lac_-yhjH* group. Varying degrees of joint adhesion and fibrin depositions between the joint cavity and synovial membrane in the abovementioned groups. **(D)** In the LB control group, there were no significant changes in the right joint cavity. **(E)** In the talc control group, there was severe aseptic inflammation. **(F)** The arthritis index of each group. Results are represented as the mean ± SD. ***p < 0.001 versus LB and talc control groups (control group). ANOVA test was used to compare group differences (600 × 600 DPI). LB, Luria–Bertani; talc, magnesium tetrasilicate.

From the second day after surgery, the rabbits of all experimental groups presented joint swelling, pain, redness, flexion contracture, and progressive lameness. After surgery, there were varying degrees of fibrin depositions and mucus containing white pus in the knee joint cavity of the infection groups, and the soft tissue also showed varying degrees of hyperemia (**Figures 1A–C**). We identified fibrin depositions using Gram stain and found that they were positive for *P. aeruginosa*, while the LB control group (**Figure 1D**) and the talc control group (**Figure 1E**) presented no such symptoms or signs. Interestingly, the arthritis index scores of the groups were different. The PAO1*ΔwspF* group had achieved the highest clinical score. A statistically significant difference was found among all the groups (p < 0.05) (**Figure 1F**).

### Comparison of c-di-GMP Content Measurement and Biofilm Formation *In Vitro*

**Figure 2:**
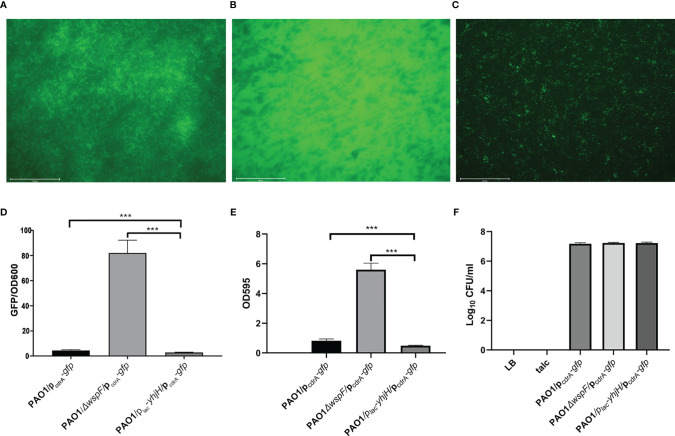
Comparison of c-di-GMP content measurement, biofilm formation *in vitro*, and the fibrinous deposition colony count *in vivo*. **(A)** The expression of p_cdrA_ *-gfp* in biofilm in the PAO1 group. **(B)** The expression of p_cdrA_ *-gfp* in biofilm in the PAO1*ΔwspF* group. **(C)** The expression of p_cdrA_ *-gfp* in biofilm in the PAO1/p*_lac_-yhjH* group. **(D)** Comparison of c-di-GMP content measurement in the three infection groups. The optical densities of 600 nm (OD600) and green fluorescent protein (GFP) fluorescence (in relative fluorescence units) (emission wavelength 535 nm; excitation wavelength 485 nm) were recorded. **(E)** Comparison of biofilm formation in the three infection groups. **(F)** Comparison of the fibrinous deposition colony count *in vivo*. Results are represented as the mean ± SD. ***p < 0.001. ANOVA test was used to compare group differences. (600 × 600 DPI). LB, Luria–Bertani; talc, magnesium tetrasilicate.

The expression of p_cdrA_ *-gfp* in biofilm was different in different groups (**Figures 2A–C**). The biofilms formed by PAO1*ΔwspF*/p_cdrA_ *-gfp* were the most (**Figure 2B**) and PAO1*/p_lac_-yhjH*/p_cdrA_ *-gfp* were the least (**Figure 2C**). PAO1*ΔwspF*/p_cdrA_ *-gfp* contained the most c-di-GMP and the least PAO1*/p_lac_-yhjH*/p_cdrA_ *-gfp* strains. The comparison of PAO1*ΔwspF*/p_cdrA_ *-gfp*, PAO1/p_cdrA_ *-gfp*, and PAO1*/p_lac_-yhjH*/p_cdrA_ *-gfp* was statistically significant (**Figure 2D**). Similarly, the biofilms formed by PAO1*ΔwspF*/p_cdrA_ *-gfp* were the most and PAO1*/p_lac_-yhjH*/p_cdrA_ *-gfp* were the least. The comparison of PAO1*ΔwspF*/p_cdrA_ *-gfp*, PAO1/p_cdrA_ *-gfp* and PAO1*/p_lac_-yhjH*/p_cdrA_ *-gfp* was also statistically significant (**Figure 2E**).

### Results of Tissue Colony Count

The fibrinous deposition colony count of the three infection groups was almost the same (results are represented as the mean ± SD. The PAO1 group: 1.51 × 10^7^ ± 0.26, the PAO1*△wspF* group: 1.71 × 10^7^ ± 0.15, and the PAO1*/p_lac_-yhjH* group: 1.69 × 10^7^ ± 0.25), and there was no significant difference (**Figure 2F**).

### Histopathological Results

**Figure 3:**
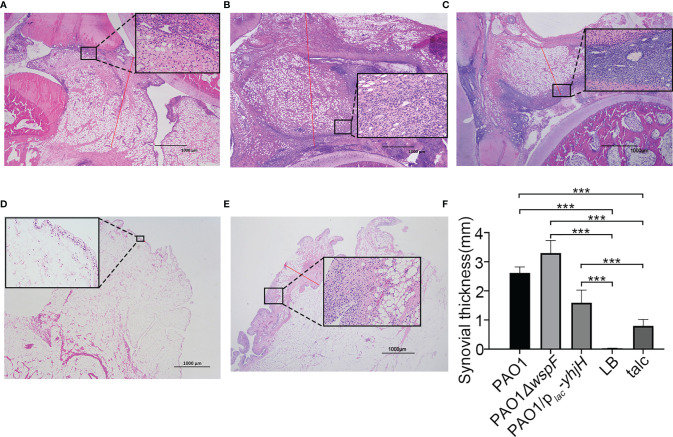
The morphological changes of the synovial membrane 7 days after injection. **(A)** Thickening of the synovial membrane with inflammatory cell infiltration in the PAO1 infection group. The length of the red line represents the measured synovial thickness (H&E staining, ×20). Morphological changes of the synovial membrane marked and enlarged with a black box (H&E staining, ×200). **(B)** Infiltration of a marked amount of inflammatory cells resulted in significant thickening of the synovial membrane in the PAO1*ΔwspF* group than that in the PAO1 group. **(C)** The synovial membrane was slightly thickened with only mild inflammation in the PAO1/p*_lac_-yhjH* group. **(D)** Fewer inflammatory cells present in the LB control group. **(E)** Severe aseptic inflammation in the talc control group. **(F)** The synovial membrane thickness of each group. Results displayed as the mean ± SD. ***p < 0.001 versus LB and talc control groups (control group). ANOVA test was used to compare group differences (600 × 600 DPI). LB, Luria–Bertani; talc, magnesium tetrasilicate.

There were significant differences in H&E-stained sections of rabbit joint samples in all groups (**Figures 3A–E**). In the infection groups, varying degrees of inflammatory cell infiltration were observed in the synovial membrane (**Figures 3A–C**). In the talc control groups, mild inflammatory cell infiltration was also observed because of an aseptic inflammatory reaction (**Figure 3E**). Neutrophils were detected in the synovial membrane (**Figures 3A–C, E**). Surprisingly, the synovial membrane of the experimental groups was significantly thickened (*p* < 0.05). There was a statistically significant difference in the synovial membrane thickness in these groups. (PAO1*△wspF* > PAO1 > PAO1*/p_lac_-yhjH* > talc control group > LB control group) (*p* < 0.05) (**Figure 3F**).

### Scanning Electron Microscopy Features

**Figure 4:**
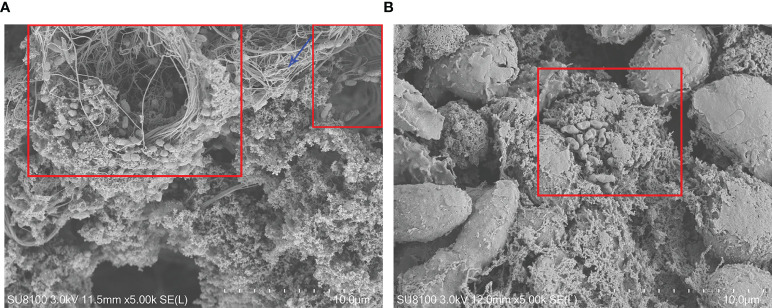
Fibrin depositions and the synovial membrane surface by scanning electron microscopy 7 days after inoculation. **(A)** Biofilm structures on the synovial membrane surface (×5,000) (**B**). Biofilm structures in the fibrin depositions (×5,000). PAO1 wild-type strains were embedded in electron-dense extracellular matrix (red box), which appeared to be biofilm structures. The blue arrow indicates a fibrin structure.

The mushroom-like structures representative of mature biofilms could be observed on the surface of the synovial membrane by microorganisms adhering to the cell surfaces. Microorganisms were embedded in a self-produced extracellular matrix (**Figures 4A, B**). Conversely, this structure was not observed on the surface of the synovial membrane in the control groups.

### PNA-FISH

**Figure 5:**
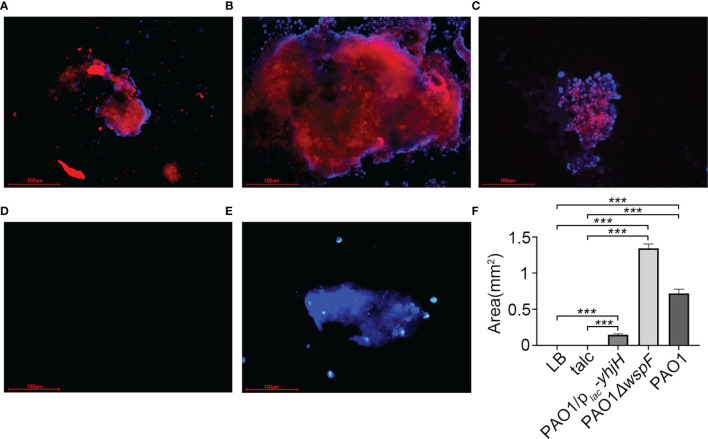
The PNA-FISH of fibrinous deposition in the purulent exudate 7 days after inoculation. The PNA-FISH kit used a *Pseudomonas aeruginosa*-specific probe (red) and a non-specific nucleic acid stain DAPI (blue) to identify biofilms (×40). The figure showed that the bacteria were surrounded by host cells in **(A–C)**. In red the aggregate, and in blue the polymorphonuclear leukocytes surrounding the aggregate. **(A)** PAO1 group. **(B)** PAO1*ΔwspF* group. **(C)** PAO1/p*_lac_-yhjH* group. **(D)** LB control group. **(E)** Talc control group. **(F)** Biofilm size measured by PNA-FISH. Results displayed as the mean ± SD. ***p < 0.001 versus LB and talc control groups (control group), and the differences among the infection groups were statistically significant. ANOVA test was used to compare group differences (600 × 600 DPI). PNA-FISH, peptide nucleic acid fluorescence *in situ* hybridization; DAPI, 4,6-diamino-2-phenylindole; LB, Luria–Bertani; talc, magnesium tetrasilicate.

PNA probes can specifically bind to the RNA sequences of the *P. aeruginosa* ribosome to form RNA-PNA hybrid complexes that are exceptionally resistant to heat and physical and chemical factors. When the RNA–PNA hybrid complexes are observed through a fluorescence microscope, they are marked as a red fluorescence to identify biofilm aggregates in fibrinous depositions of purulent exudate. Red fluorescence surrounded by blue fluorescence could be seen in the three infection groups, indicating biofilm aggregates embedded in a matrix of extracellular polymeric substances (**Figures 5A–C**). However, there was no purulent exudate in the LB control group (**Figure 5D**). In the talc control group, there was no red fluorescence, only blue fluorescence (**Figure 5E**). Interestingly, we found that the amount of biofilm formation was different in each of the infection groups. The greatest amount of biofilm was detected in the PAO1*△wspF* group, while the amount of biofilm in the PAO1*/p_lac_-yhjH* group was the least; these differences between groups were statistically significant (**Figure 5F**).

These results suggested that we had successfully established a rabbit model for septic knee arthritis with biofilm formation by *P. aeruginosa*. In addition, this model implied that the amount of formed biofilms may be related to the intracellular c-di-GMP signaling pathway.

## Discussion

We investigated the formation of biofilms in a septic knee arthritis rabbit model infected with *P. aeruginosa*. Three *P. aeruginosa* strains with different expression of cellular c-di-GMP levels were used to explore whether the mechanism of biofilm formation was related to c-di-GMP signaling pathways. To demonstrate the above hypothesis, we established a novel rabbit model for septic knee arthritis infected with *P. aeruginosa*. Based on the mutual support of macroscopic appearance, histopathological results, and bacterial culture, we confirmed that a rabbit model for septic knee arthritis had been successfully established. Furthermore, we observed the fibrinous deposition of purulent exudate using SEM and PNA-FISH and quantitatively analyzed the fibrinous deposition of purulent exudate using PNA FISH. Our results showed that *P. aeruginosa* with a higher intracellular c-di-GMP level promoted biofilm formation.

The establishment of an animal model of biofilm infection is of great significance for exploring the mechanisms involved in biofilm formation. To date, there has been no animal model for knee septic arthritis induced by *P. aeruginosa* biofilms. Sinha et al. (2019), Marcheix et al. (2018), and Oner et al. (2011) selected New Zealand white rabbits to establish an animal model of joint infection and used this model to assess the therapeutic effects of antibiotics on joint infection. Our laboratory also used this animal model to verify the establishment of a joint infection model. However, our study was the first to observe biofilm formation in a rabbit joint infection model. To establish the infection model, 10^6^ CFU of *P. aeruginosa* (2 ml) was injected into the rabbit’s knee joint cavity. We used three *P. aeruginosa* strains carrying different intracellular c-di-GMP levels to establish models and prevent insufficient virulence of the bacteria The results showed that the injection of *P. aeruginosa* into the joint cavity could form biofilms. The animals were euthanized 7 days after inoculation, and a detailed macroscopic analysis revealed that there were numerous inflammatory exudates in the knee joint cavity, and biofilms were visible in all three experimental groups. Histological analysis in the H&E-stained section revealed an increased accumulation of immune cells in the synovial membrane, including lymphocytes and neutrophils, which are considered to be the most important predictor of inflammation. Compared with the mouse animal model (Jin et al., 2019), New Zealand white rabbits are larger in size and reduce the influence of individual animal differences.

SEM can be applied to observe biofilms (Wi and Patel, 2018). We observed the morphology of the biofilm formed by *P. aeruginosa* under SEM. The SEM used an electron beam and electron lens instead of a light beam and optical lens, and the magnification is thousands of times greater. The scanning electron microscopic features of biofilms are as follows: smaller colonies at higher magnification, three-dimensional structure, and the bacteria embedded in a compact fibrous material with disordered cilia arrangement (Ramadan et al., 2005). Other similar studies (Saraswathi and Beuerman, 2015; Zhang et al., 2020) have also reported the observation of biofilms under SEM, but not in the joint cavity. Planktonic bacteria scattered beneath the mushroom-like structures are an indication that these are more likely to derive from the mature biofilm. The disadvantage of this model is that only the approximate surface morphology of the biofilm image can be observed, as the three-dimensional structure of the biofilm cannot be observed due to the limitations of SEM. The experimental design needs to be improved in the future.

We also visualized the bacterial populations in the biofilms using PNA-FISH. FISH techniques have the advantage of identifying the specific bacteria that produce the biofilm substrate (Sanderson et al., 2006). The application of PNA-FISH can offer quantitative analysis and a better understanding of biofilm structures in fibrinous depositions of purulent exudate. Wei et al. (2019) reported that insulin therapy promoted the formation of *P. aeruginosa* biofilm by increasing intracellular c-di-GMP levels as detected by PNA-FISH and led to chronic wound infection and delayed wound healing. Furthermore, Zhang et al. (2020) reported that the amount of biofilm formation of *P. aeruginosa* strains with different intracellular c-di-GMP levels could be compared using PNA-FISH in the rabbit empyema model. Similar PNA-FISH images of biofilms on wound tissues and on the surface of the pleura have been described (Wei et al., 2019; Zhang et al., 2020). The imaging technique used in this study presents advantages in microbiology studies, particularly when FISH and SEM are combined. Furthermore, this study showed that in the rabbit model for septic knee arthritis, the higher the intracellular c-di-GMP levels, the more biofilms could be detected, while fewer biofilms were observed in the low intracellular c-di-GMP levels group.

Intracellular c-di-GMP is an important second messenger molecule widely found in bacteria. It regulates the motility of bacteria, biofilm production, cytotoxicity, cell cycle, cell division, and other physiological and biochemical process (Valentini and Filloux, 2019). Matsumoto et al. (2021) reported that intracellular c-di-GMP levels of the *dgcS* deletion mutant (*ΔdgcS* mutant) were significantly reduced, which resulted in the loss of biofilm formation. Dawson et al. (2021) also showed that c-di-GMP enhanced early biofilm formation in *Clostridium difficile*. This study, like ours, showed that intracellular c-di-GMP levels promoted biofilm formation, although our study is the first to report biofilm formation in the joint cavity. Furthermore, our study provided evidence of biofilm formation based on macroscopic appearance and PNA-FISH.

The present study has limitations that should be considered. First, biofilm formation depends on a complex combination of several factors, including temperature and culture conditions (Høyland-Kroghsbo et al., 2018; Guła et al., 2019). Second, the sample size of our study was limited sample size. Third, although the study provides evidence that c-di-GMP is involved in the mechanism regulating biofilm formation, we did not investigate the molecular mechanism c-di-GMP in detail.

Our findings will help researchers carry out in-depth research on *P. aeruginosa* biofilms and further clarify the pathogenic and drug resistance mechanisms of *P. aeruginosa* biofilms. In the rabbit septic arthritis animal model established in this study, the higher the maturity of the synovial bacterial biofilm, the more severe the inflammation and infection of septic arthritis. Our findings suggest that patients with chronic septic arthritis who have severe infection symptoms and poor drug treatment effects may have bacterial biofilms. For such patients, drugs and treatment approaches that can effectively remove bacterial biofilms are worthy of our further exploration and research.

In conclusion, the New Zealand white rabbit infection model established in this study provides an effective method for the study of biofilms *in vivo*, which can be used to study the mechanism of biofilm formation and guide the clinical therapy of stubborn septic arthritis. This is the first study to induce *P. aeruginosa* biofilm formation in a rabbit model for septic knee arthritis. Our model will certainly find application in guiding clinical medication discovery in the future.

## Data Availability Statement

The raw data supporting the conclusions of this article will be made available by the authors, without undue reservation.

## Ethics Statement

The animal study was reviewed and approved by the Medical Ethics Committee of the First Affiliated Hospital of Guangxi Medical University (no. 202007001).

## Author Contributions

Conceptualization: KW and QW. Data curation: KW and QW. Formal analysis: DL, LZ, and JL. Investigation: DL, LZ, JL, and WD. Methodology: DL, LZ, KW, and QW. Project administration: KW and QW. Resources: DL, LZ, JL, and WD. Software: DL, LZ, JL, and WD. Supervision: KW and QW. Validation: KW and QW. Visualization: DL, KW, and QW. Writing—original draft: DL, KW, and QW. Writing—review and editing: DL, KW, and QW. All authors contributed to the article and approved the submitted version.

## Funding

The work was supported by the National Natural Scientific Funds (81760024).

## Conflict of Interest

The authors declare that the research was conducted in the absence of any commercial or financial relationships that could be construed as a potential conflict of interest.

## Publisher’s Note

All claims expressed in this article are solely those of the authors and do not necessarily represent those of their affiliated organizations, or those of the publisher, the editors and the reviewers. Any product that may be evaluated in this article, or claim that may be made by its manufacturer, is not guaranteed or endorsed by the publisher.
