# Supplementary material for: Biofilm Formation by Pseudomonas aeruginosa in a Novel Septic Arthritis Model
Source: Front Cell Infect Microbiol. 2021 Sep 21;11:724113. doi: 10.3389/fcimb.2021.724113 (PMC8490669; doi:10.3389/fcimb.2021.724113)
Supplement: Supplementary file 2 [file Table_1.docx]

| **Table 1 Body weight of all experimental rabbit groups before surgery.** | | | | | |
| --- | --- | --- | --- | --- | --- |
| **Baseline**  **parameter** | **PAO1 group**  **(n = 4)** | **PAO1*ΔwspF* (n = 4)** | **PAO1*/p_lac_-yhjH***  **(n = 4)** | **LB**  **(n = 4)** | **Talc**  **(n = 4)** |
| **Body weight (kg)** | 2.16 ± 0.07 | 2.18 ± 0.08 | 2.23 ± 0.04 | 2.2 ± 0.08 | 2.2 ± 0.08 |
| All results are represented as mean ± standard deviation(SD). LB, Luria-Bertani; talc, magnesium tetrasilicate. | | | | | |
